# Disproportionality analysis of adverse events in advanced lung cancer treated with atezolizumab plus platinum-based combination chemotherapy

**DOI:** 10.1186/s40780-025-00522-6

**Published:** 2025-12-06

**Authors:** Katsuyuki Hazama, Toru Imai, Naohiro Tochikura, Shinsaku Washinosu, Susumu Ootsuka, Kazuhiko Hanada

**Affiliations:** 1 Department of Pharmacy, Nihon University Itabashi Hospital, Itabashi-ku, Tokyo, 173-8610 Japan; 2 Department of Pharmacometrics and Pharmacokinetics, Meiji Pharmaceutical University, 2-522-1 Noshio, Kiyose, Tokyo, 204-8588 Japan

**Keywords:** FDA adverse event reporting system, Advanced non-squamous non-small cell lung cancer, Atezolizumab

## Abstract

**Background:**

Advanced non-squamous non-small cell lung cancer has a poor prognosis, and immune checkpoint inhibitors (ICIs) plus platinum-based combination chemotherapy is the first-line treatment. Atezolizumab, an ICI, has three regimens: atezolizumab + carboplatin (CBDCA) + paclitaxel + bevacizumab [ABCP], atezolizumab + CBDCA + nab-paclitaxel (nab-PTX) [A + CnP], and atezolizumab + CBDCA + pemetrexed [A + CbP]. However, clinical trials directly comparing these regimens have not been reported, and the differences in adverse events have not been fully clarified.

**Methods:**

We calculated the reporting odds ratio (ROR), a measure of adverse drug reaction (ADR) signals, using the FDA Adverse Event Reporting System (FAERS) to compare the major ADRs of ABCP, A + CnP, and A + CbP regimens.

**Results:**

The ROR (95% confidence interval [CI]) for rash and hypersensitivity-related adverse events was 2.06 (1.81–2.34) for ABCP, 0.81 (0.42–1.56) for A + CnP, and 0.57 (0.37–0.87) for A + CbP; the signal was only detected for the ABCP regimen. In acute kidney injury (AKI), it was ABCP: 1.24 (0.89–1.73), A + CnP: 1.16 (0.37–3.60), and A + CbP: 1.76 (1.06–2.93), and signal was detected only for the A + CbP regimen. Contrarily, signals were detected for colitis, drug-induced liver injury, and pneumonitis for all regimens.

**Conclusions:**

Rash and hypersensitivity-related adverse events and AKI were more frequently reported with the ABCP and A + CbP regimens, respectively. These observations may help generate hypotheses regarding regimen-specific adverse event profiles and support future studies toward individualized chemotherapy.

**Supplementary Information:**

The online version contains supplementary material available at 10.1186/s40780-025-00522-6.

## Background

Lung cancer is one of the deadliest cancers in the world. According to World Health Organization estimates, more than 1.8 million people worldwide died of lung cancer in 2021 [1]. Lung cancer is histologically divided into small cell lung cancer and non-small cell lung cancer (NSCLC). Approximately 85% of all lung cancers are NSCLCs, and more than 50% are non-squamous NSCLCs (non-Sq NSCLC) [2, 3]. Advanced non-Sq NSCLC has a poor prognosis, and chemotherapy is the first-line treatment. For decades, platinum-based combination chemotherapy with paclitaxel (PTX) or pemetrexed (PMX), which are third-generation cytotoxic anticancer drugs, has been the standard of care. However, its efficacy has been poor, with an overall survival (OS) of less than 12 months [4]. Recently, immune checkpoint inhibitors (ICIs), which have a different mechanism of action from that of conventional anticancer drugs, have attracted attention. ICIs inhibit the binding of programmed cell death protein 1 (PD-1) and programmed death ligand 1 (PD-L1) / PD-L2 expressed on T cells and activate them by blocking the transmission of immunosuppressive functions, resulting in anti-tumor effects [5]. A phase III trial on advanced non-Sq NSCLC reported a significant increase in OS and progression-free survival with the addition of an ICI to platinum-based combination chemotherapy [6–8]. ICIs include PD-1 and PD-L1 inhibitors, and it has been reported that there is no significant difference in efficacy and safety between the two types of drugs [9]. In contrast, atezolizumab, a PD-L1 inhibitor, does not act on PD-1/PD-L2, which may minimize its effect on immune homeostasis and has shown efficacy in NSCLC with or without PD-L1 expression [10]. Currently, atezolizumab combination regimens, including atezolizumab + carboplatin (CBDCA) + PTX + bevacizumab (Bev) [ABCP], atezolizumab + CBDCA + nab-paclitaxel (nab-PTX) [A + CnP], and atezolizumab + CBDCA + pemetrexed (PMX) [A + CbP], are listed as first-line regimens [6–8]. The selection of treatment is based on the patient’s performance status and general condition, including disease status and lung function, along with the adverse events of chemotherapy, to make a comprehensive decision on whether the patient can tolerate the treatment. However, the OS in the phase III trial was reported to be 19.5 months for ABCP, 17.5 months for A + CnP, and 18.6 months for A + CbP, making these regimens almost equivalent in terms of efficacy [6–8]. There were no significant differences in the side effects reported in these clinical trials, and there were no criteria to judge which chemotherapy should be selected. Furthermore, details of the adverse event profile of atezolizumab in combination with platinum-based chemotherapy in clinical practice have not been fully clarified. Although randomized controlled trials (RCT) are the most reliable method to explore the adverse effects of drugs, they are time-consuming, expensive, and labor-intensive. Therefore, they are difficult to conduct in practice. Recently, studies have been conducted on drug reactions using the FDA Adverse Event Reporting System (FAERS) of the United States Food and Drug Administration (FDA), a large spontaneous reporting system database of adverse drug reactions (ADRs) [11, 12]. The FAERS is the largest adverse event reporting database in the world, containing over 16 million reports, including data from patients who are often excluded from clinical trials. Therefore, it can complement clinical trial data and provide information on a broader and more diverse patient population.

Therefore, in this study, with the aim of generating hypotheses for selecting chemotherapy suitable for patients’ backgrounds, we compared the major adverse events that interfere with treatment continuation—namely, injuries of the skin, liver, lungs, and gastrointestinal and renal systems—among ABCP, A + CnP, and A + CbP regimens using the FAERS.

## Methods

### Data acquisition and preprocessing

The FAERS data were downloaded from the FDA website (http://www.fda.gov/). The FAERS database contains seven data tables: “DEMO,” “DRUG,” “REAC,” “OUTC,” “RPSR,” “THER,” and “INDI.” The following files were used in this study: DEMO, DRUG, and REAC. The DEMO file contains basic patient information, such as age, and date and country of adverse event; the DRUG file contains the drug name, route of administration, and dose; the REAC file contains the name of the adverse event. Navicat for SQLite version 16.1.11 was used to create the FAERS dataset used in this study. In duplicate reports, only the most recent adverse event was analyzed as per the FDA recommendation.

### FAERS data preprocessing and case selection

As a preprocessing step for the FAERS data, duplicate reports were removed, reducing the total number of cases from 35,777,597 to 28,136,819. Cases under 20 and over 100 years of age were excluded, as atezolizumab combination therapy for advanced NSCLC is not approved for pediatric patients, and cases over 100 years are likely to be miscoded. Drug names were standardized using the DrugBank database, and unmatched drugs were excluded as erroneous reports. The mean ages (± SD) of the analyzed cases were 62.1 ± 10.8 for ABCP, 64.7 ± 16.7 for A + CnP, and 65.4 ± 10.1 for A + CbP.

The analysis population was extracted from the entire FAERS database, selecting cases that included the drugs of interest. All drug fields in the DRUG file (Primary suspected, Secondary suspected, Concomitant, and Interacting) were utilized. Combination regimens were identified as follows: first, cases including atezolizumab were extracted, followed by carboplatin, and then nab-paclitaxel, pemetrexed, or paclitaxel, and bevacizumab sequentially, to construct the ABCP, A + CnP, and A + CbP regimens. The analysis was limited to cases reported from 2018 onward, after the approval of atezolizumab combination therapy for advanced NSCLC in the United States.

### Database search

We accessed and downloaded all reports added to the FAERS from January 1, 2018, to March 30, 2024. Preferred terms (PTs) selected for analysis were based on the ICH International Medical Dictionary for Regulatory Activities (MedDRA ver. 26.0), and included those related to rash and hypersensitivity-related adverse events - anaphylactic reaction (PT 10002198), urticaria (PT 10046735), drug hypersensitivity (PT 10013700), erythema multiforme (PT 10015218), Stevens-Johnson syndrome (PT 10042033), toxic epidermal necrolysis (PT 10044223), and rash (PT 10037844) - as well as colitis (PT 10009887), acute kidney injury (PT 10069339), drug-induced liver injury (PT 10072268), and pneumonitis (PT 10035742). Regarding hematologic adverse events, signal detection analyses were also performed. Signals were observed across all regimens, with no apparent differences between them (Supplementary Table S1). Since the present study aimed to compare organ-specific adverse event profiles among regimens, hematologic toxicities—which are commonly observed across most regimens containing cytotoxic agents—were deemed unsuitable for comparative evaluation. Therefore, hematologic adverse events were excluded from the primary analysis, and the results are provided in the Supplementary Table to maintain analytical transparency.

### Statistical analyses

The ROR is a widely used method in adverse drug event signal detection. It has been extensively employed in studies based on FAERS data [11, 12]. When the lower limit of the 95% CI for the ROR was >1, the adverse event was considered to have a significantly frequent occurrence with the use of the drug of interest compared with the use of all other drugs. The ROR score was defined as $\:\frac{n11/n12}{n21/n22}=\frac{n11\:\times\:\:n22}{n12\:\times\:\:n21}\:$ (Table 1). Considering rash and hypersensitivity-related adverse events from the ABCP regimen as an example, “n11” is the number of safety reports of patients who received the ABCP regimen and developed rash and hypersensitivity-related adverse events, and ‘’n12’’ is the number of safety reports of patients who received the ABCP regimen and reported adverse events other than rash and hypersensitivity-related adverse events. ‘’n21’’ is the number of safety reports in which the patient did not receive the ABCP regimen and developed rash, and ‘’n22’’ is the number of safety reports in which the patient did not receive the ABCP regimen and reported adverse events other than rash and hypersensitivity-related adverse events.

**Table 1:** Two-by-two table used for the calculation of reporting odds ratios

		Suspected event reported	No suspected event reported	Total
Medication	Index group	n_11_	n_12_	n_1+_
	Reference group	n_21_	n_22_	n_2+_
		n_+ 1_	n_+ 2_	n_++_

$$\begin{aligned}\:\mathrm{ROR\:=\:}\frac{\mathrm{(n}_\mathrm{11}\mathrm{\:/\:n}_\mathrm{12})}{\mathrm{(n}_\mathrm{21}\mathrm{\:/\:n}_\mathrm{22})}\mathrm{=\:}\frac{\mathrm{(n}_\mathrm{11}\times\mathrm{n}_\mathrm{22})}{\mathrm{(n}_\mathrm{12}\times\mathrm{n}_\mathrm{21})}\:\:\:\:\:\mathrm{95\%CI\:=\:}{\mathrm{e}}^{\mathrm{ln}\text{}\left(\mathrm{ROR}\right)\:\:\mathrm{1.96\:}\sqrt{\frac{\mathrm{1}}{\mathrm{n}_\mathrm{11}}\mathrm{\:+\:}\frac{\mathrm{1}}{\mathrm{n}_\mathrm{12}}\mathrm{\:+\:}\frac{\mathrm{1}}{\mathrm{n}_\mathrm{21}}\mathrm{\:+\:}\frac{\mathrm{1}}{\mathrm{n}_\mathrm{22}}}}\end{aligned}$$

### Data source

In this study, the FAERS was selected as the data source to evaluate adverse event trends associated with atezolizumab combination chemotherapy regimens. The FAERS contains over 16 million adverse event reports from multiple countries, including a substantial number of oncology- and ICI-related cases, enabling sufficient statistical power to detect potential associations between specific regimens and adverse events.

In contrast, the Japanese Adverse Drug Event Report database is limited to domestic reports in Japan (70,000–80,000 cases per year), which restricts its ability to capture global drug-use patterns and diverse patient backgrounds. Although VigiBase is a large international database, its restricted access poses challenges in ensuring analytical reproducibility. The FAERS, being fully publicly available, provides advantages in transparency, reproducibility, and independent verification of analyses.

Nevertheless, the FAERS shares inherent limitations of spontaneous reporting systems, such as reporting bias, heterogeneity of information, and missing data. In this study, we applied data-cleaning procedures, including the exclusion of duplicate records, to minimize their potential impact. Considering its large case volume, international diversity, and reproducibility, the FAERS was deemed the most appropriate data source for this study.

## Results

### Reporting odds ratio (ROR) for each adverse event

**Fig. 1:**
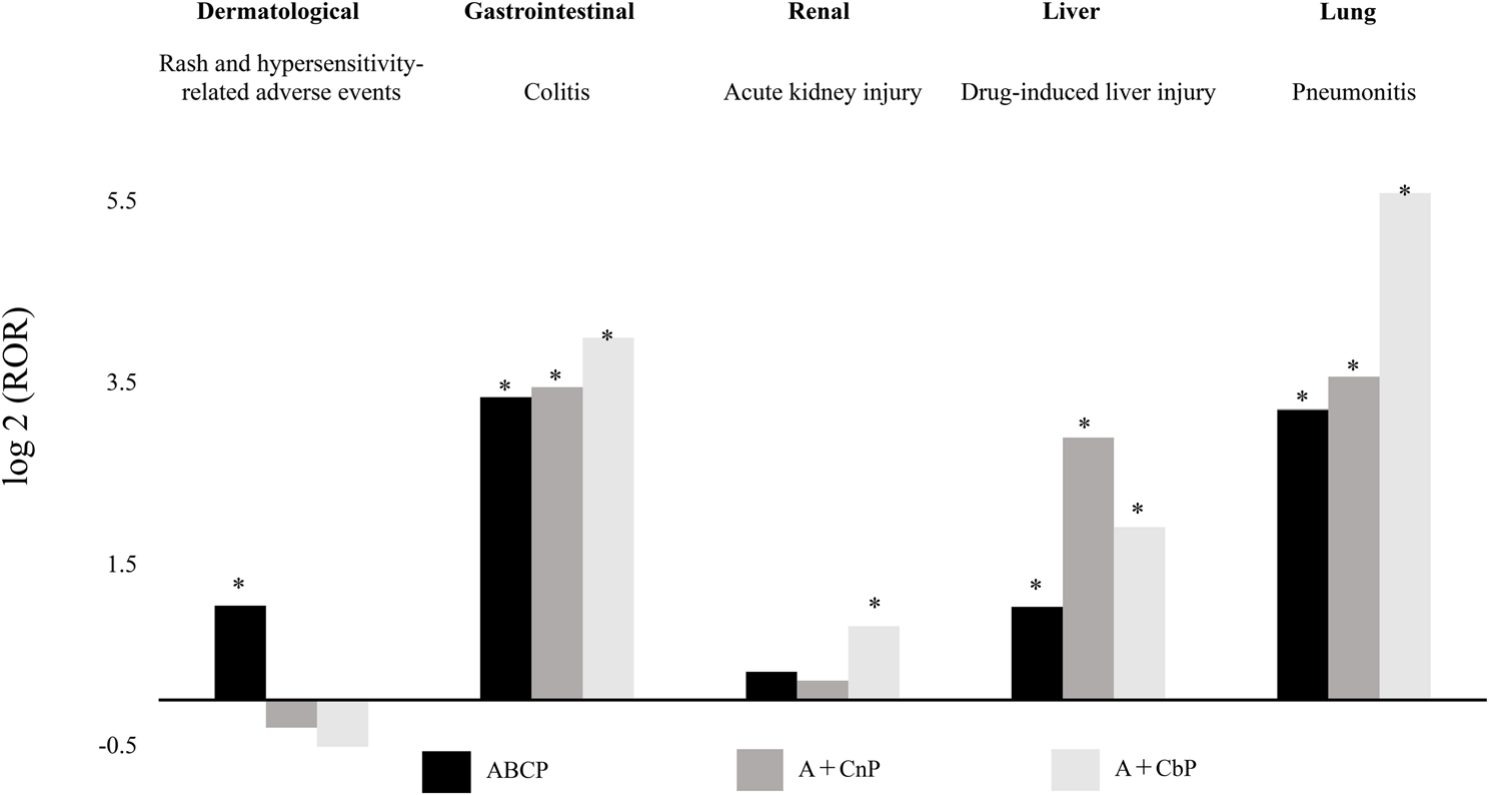
Comparison of adverse events reported in ABCP, A + CnP, and A + CbP regimen groups. The RORs identified for each adverse events were compared. For ease of data analysis and interpretation, the data are presented on a logarithmic scale. The detected signals are denoted as [*]. ABCP : Atezolizumab + CBDCA + PTX + Bev. A + CnP : Atezolizumab + CBDCA + nab-PTX. A + CbP : Atezolizumab + CBDCA + PMX

The ROR estimates for ABCP, A + CnP, and A + CbP for adverse events detected as signals are shown in Table 2. To facilitate data analysis and interpretation, the RORs of each adverse event were compared: the RORs of ABCP, A + CnP, and A + CbP are shown and expressed as an increase or decrease on a logarithmic scale (Fig. 1). The ROR (95% confidence interval [CI]) for rash and hypersensitivity-related adverse events was 2.06 (1.81–2.34) for ABCP, 0.81 (0.42–1.56) for A + CnP, and 0.57 (0.37–0.87) for A + CbP. (Supplementary Table S2). The lower limit of the 95% CI of the ROR for ABCP was > 1, and a signal was detected, whereas no signal was detected for A + CnP and A + CbP. In colitis, the RORs were as follows: ABCP: 10.1 (7.61–13.3), A + CnP: 10.9 (4.54–26.4), A + CbP: 15.9 (10.7–23.9), with signals being detected in all regimens. For acute kidney injury (AKI), the findings were 1.24 (0.89–1.73) for ABCP, 1.16 (0.37–3.60) for A + CnP, and 1.76 (1.06–2.93) for A + CbP. The lower limit of the 95% CI of the ROR for A + CbP was > 1, and a signal was detected, whereas no signal was detected for ABCP and A + CnP. Drug-induced liver injury (DILI) showed the following RORs: ABCP: 2.04 (1.06–3.93), A + CnP: 7.44 (2.39–23.2), and A + CbP: 3.75 (1.56–9.03), and all signals were detected. In pneumonitis, the findings were ABCP: 9.20 (6.56–12.9), A + CnP: 11.8 (4.41–31.5), and A + CbP: 48.1 (36.6–63.2), with signals being detected for all regimens.

**Table 2:** Number of cases and ROR (95% CI) of adverse events for ABCP, A + CnP, and A + CbP regimens reported in FAERS

Adverse event	ABCP			A + CnP			A + CbP		
	Case	ROR	95% CI	Case	ROR	95% CI	Case	ROR	95% CI
Rash and hypersensitivity-related adverse events	245	2.06*	1.81–2.34	9	0.81	0.42–1.56	21	0.57	0.37–0.87
Colitis	50	10.1*	7.61–13.3	5	10.9*	4.54–26.4	24	15.9*	10.7–23.9
Acute kidney injury	35	1.24	0.89–1.73	3	1.16	0.37–3.60	15	1.76*	1.06–2.93
Drug-induced liver injury	9	2.04*	1.06–3.93	3	7.44*	2.39–23.2	5	3.75*	1.56–9.03
Pneumonitis	34	9.20*	6.56–12.9	4	11.8*	4.41–31.5	53	48.1*	36.6–63.2

Signals detected are marked with [*]

ABCP: Atezolizumab + CBDCA + PTX + Bev. A + CnP: Atezolizumab + CBDCA + nab-PTX. A + CbP: Atezolizumab + CBDCA + PMX

## Discussion

In this study, we analyzed the adverse events associated with ABCP, A + CnP, and A + CbP. Rash and hypersensitivity-related adverse events were reported with ABCP, and AKI was reported with A + CbP, suggesting differences in adverse event profiles among the regimens. For chemotherapies without direct comparisons, analysis using the FAERS is expected to clarify the differences in adverse events and can lead to the selection of drug therapy appropriate for the patient’s background [11, 12]. Meirson et al. reported differences in serious adverse events between three treatments: vemurafenib + cobimetinib, dabrafenib + trametinib, and encorafenib + binimetinib [11]. Matsumoto et al. also reported differences in adverse events between the axitinib + pembrolizumab and lenvatinib + pembrolizumab regimens [12].

ICIs or cytotoxic chemotherapy are known to cause various adverse events such as injuries of skin, liver, lung, and gastrointestinal and renal systems [13, 14]. Rash and hypersensitivity-related adverse events were analyzed by integrating several relevant PTs (anaphylactic reaction, urticaria, drug hypersensitivity, erythema multiforme, Stevens-Johnson syndrome, toxic epidermal necrolysis, and rash). Among these, rash was the most frequently reported event, and signals were detected only with the ABCP regimen. It has been suggested that the increased secretion of inflammatory cytokines and production of autoantibodies by the activation of T and B cells are the mechanisms involved in the pathogenesis of rash caused by ICIs [15]. In contrast, histamine-mediated skin rashes have been reported to occur with cytotoxic chemotherapy because of additives such as solubilizers and emulsifiers in the dosage form [16]. PTX in ABCP contains Cremophor EL as an additive, which has been reported to cause the release of histamine from mast cells and subsequent skin rash [16]. Bev also contains a polysorbate that has histamine-releasing effects [17, 18]. In contrast, the A + CnP and A + CbP combination regimens, CBDCA, nab-PTX, and PMX do not contain additive agents that have been reported to have histamine-releasing effects. Furthermore, previous studies have reported that the concomitant use of ICIs with drugs known to induce skin eruptions may increase the incidence of rashes [15]; a similar trend was observed in the ABCP regimen. In this study, a relatively high reporting frequency of rash and hypersensitivity-related adverse events was observed for ABCP in the FAERS database. Although the underlying mechanism remains to be elucidated, one possible hypothesis is that patients with histamine-related conditions, such as seasonal allergic rhinitis, allergic asthma, or intestinal disorders (including Crohn’s disease and ulcerative colitis), who are known to have higher plasma histamine concentrations than healthy individuals, may be more susceptible to ABCP-related skin eruptions [19, 20]. Skin disorders, including rash, may be associated with allergic or infusion-related reactions, which can, in some cases, lead to treatment interruption or discontinuation. Therefore, premedication and, when necessary, switching to alternative agents may be considered. Further prospective studies are warranted to confirm this potential association.

Colitis is a major side effect of gastrointestinal injury that may require discontinuation of chemotherapy [21]. Although none of the studies have reported the incidence of colitis on the administration of atezolizumab in combination with cytotoxic chemotherapy, a signal was detected for all three regimens in our study. Atezolizumab-induced colitis is associated with T-cell activation [22]. In contrast, CBDCA, PTX, and PMX cause neutropenic enterocolitis by reducing the immune defense, damaging mucous membranes, and disrupting the intestinal microbiota [23–25].

Thus, ICIs and cytotoxic chemotherapy may be involved in colitis through different mechanisms, with a higher reporting frequency of colitis being observed when these agents were used in combination. Although the underlying mechanism remains unclear, this finding suggests a potential pharmacological interaction that warrants further investigation.

Renal injury may cause unexpected side effects due to delayed drug excretion. In this study, we analyzed AKI, a widely used indicator of acute nephrotoxicity induced by chemotherapy [26]. Therefore, AKI signals were observed only in the A + CbP group.

In a phase III trial comparing atezolizumab and docetaxel, only one (0.2%) case of renal injury resulted in the discontinuation of treatment [27]. Similarly, in previous case reports, the frequency of AKI caused by atezolizumab was low [28, 29]. In this analysis, AKI signals were not detected in the ABCP and A + CnP regimens. The findings suggest that additive renal injury resulting from the combination of atezolizumab and cytotoxic chemotherapy was not observed, and that this signal may be primarily associated with the concomitant platinum-based agents. PMX is a renally excreted drug, and patients with impaired renal function are at a greater risk of increased blood concentrations owing to delayed excretion [30]. In addition, some deaths due to renal toxicity have been reported in a blood concentration-dependent manner [31, 32]. Therefore, the selection criteria for patients in a phase III clinical trial of A + CbP were patients with relatively preserved renal function, resulting in the discontinuation of treatment in only four (1.4%) AKI cases [7]. In contrast, epidemiological studies have reported that approximately 25% of patients with lung cancer have a creatinine clearance less than 60 mL/min [33]. Therefore, it is assumed that a few patients with renal dysfunction are included in the FAERS, a spontaneous reporting system database of adverse event reports. Analysis of the FAERS dataset used in this study (28,136,819 reports in total) revealed that 271,640 reports (approximately 0.97%) were associated with PTs for renal dysfunction, suggesting that a certain number of patients with impaired renal function are included in the database. The detailed list of PTs is provided in Supplementary Table S3. The signal detected in the A + CbP regimen may reflect the contribution of elevated PMX plasma concentrations. In contrast, no signals were observed in the ABCP and A + CnP regimen groups, which may be due to the administration of CBDCA at doses adjusted for renal function using the Calvert formula. Although Bev in ABCP has been reported to cause tubular cell damage due to long-term proteinuria, it rarely leads to AKI because it is managed clinically through appropriate drug withdrawal and monitoring [34, 35]. Furthermore, PTX in ABCP and nab-PTX in A + CnP are unlikely to cause renal injury because the liver is their major metabolic pathway. Therefore, in patients with moderate renal dysfunction, careful consideration of renal function is important when selecting A + CbP. According to the KDIGO 2024 Clinical Practice Guideline for the Evaluation and Management of Chronic Kidney Disease, moderate renal impairment is defined as a creatinine clearance of 30–59 mL/min. In cases involving the A + CbP regimen among patients with moderate renal impairment, the FAERS-based disproportionality analysis showed a relatively higher reporting tendency for renal injury. However, as this study is based on observational data, these findings do not establish causality or provide specific clinical recommendations. In clinical practice, regimen selection should be based on a comprehensive assessment of renal function, overall condition, comorbidities, and concomitant medications.

Chemotherapy-induced hepatic toxicity is one of the primary causes of chemotherapeutic failure. We analyzed DILI as a chemotherapy-induced hepatic toxicity and found that a signal was detected in all regimens. Atezolizumab hepatotoxicity has been suggested to cause hepatic injury of the hepatocellular or cholestatic type owing to necrosis and inflammatory cell infiltration by the activation of cytotoxic CD8 + T cells in hepatocytes [36]. In contrast, CBDCA, PTX, and PMX may cause hepatotoxicity by direct action on hepatocytes [37]. Signals potentially related to DILI were observed in all three regimens, suggesting that atezolizumab, CBDCA, PMX, and PTX might contribute via different mechanisms. However, in some cases, chemotherapy must be discontinued when liver function is severely affected. It has been reported that in most cases, chemotherapy can be administered with a liver-protective drug at an early stage [38–40]. Therefore, it is recommended to intervene early in cases of worsening liver function and manage the patient such that treatment can be continued.

Patients with pneumonitis must discontinue chemotherapy, as it can be fatal in severe cases [41]. In this study, we analyzed pneumonitis and detected signals in all treatment regimens. The mechanism of atezolizumab-induced pneumonitis involves T-cell activation and increased autoantibodies and proinflammatory cytokines [42]. PTX and nab-PTX have been reported to be involved in type IV allergies via drug-specific T cells [41]. PMX has also been reported to cause pulmonary toxicity owing to accumulation in lung tissue and hypersensitivity pneumonitis due to hypersensitivity reactions [43, 44]. Therefore, since ICIs and cytotoxic chemotherapy may contribute to pneumonitis via different mechanisms, careful monitoring is recommended when these combination regimens are administered. In a phase III clinical trial comparing CbP and A + CbP, adverse reaction reports of pneumonitis increased from 1.5% to 3.1% [7]. Therefore, considering the possibility of pneumonitis in all three regimens, the initial symptoms of dry cough and dyspnea should be carefully monitored.

Studies of adverse events using the FAERS are valuable for providing safety information that complements RCTs, particularly because they can help identify signals of rare adverse events that are difficult to detect in RCTs and facilitate hypothesis generation [11, 12]. This study has some limitations that should be considered. As these data were obtained from observational studies and a voluntary reporting database, reporting bias is possible, and not all the adverse events may have been captured. In addition, the FAERS lacks detailed patient-level information, including medical histories, number of exposed patients, dosage, and treatment duration, which limits the scope of the analysis. Consequently, it was not possible to conduct analyses based on severity classification, quantitatively assess risk, or evaluate time-dependent trends, all of which are important for understanding the adverse effects of anticancer drugs.

Furthermore, causal relationships cannot be established from FAERS data. Some adverse events may be influenced by concomitant medications or underlying diseases; thus, the observed events may not necessarily be directly attributable to the target regimens. The FAERS is primarily based on spontaneous reports from the United States, with contributions from other countries. To this end, differences in genetic background, medical practice, drug accessibility, and reporting behavior may influence the occurrence and reporting of adverse events; therefore, caution is warranted when generalizing these findings to Japanese patients.

Considering these limitations, the results of this study should be interpreted as hypothesis-generating findings, warranting further confirmation in prospective studies.

## Conclusions

The FAERS is a valuable source of post-marketing surveillance information for drug safety research, and previous studies have reported it as a reliable source of information for adverse event analyses [11, 12]. In this study, disproportionality analyses of adverse events were conducted for the ABCP, A + CnP, and A + CbP regimen groups. The results suggested that rash and hypersensitivity-related adverse events and AKI were more frequently reported in the ABCP and A + CbP regimens, respectively, indicating that each regimen may have a distinct adverse event profile. However, because the FAERS is based on spontaneous reporting and lacks detailed clinical information, these findings should be interpreted with caution. Further clinical studies are warranted to validate the observed associations.

We hope that our findings will contribute to a better understanding of potential adverse event profiles and help support safer and more appropriate chemotherapy selection for patients with advanced non-Sq NSCLC, for whom several first-line treatment options are available.

## Supplementary Information

Below is the link to the electronic supplementary material.

Supplementary Material 1

## Data Availability

The datasets generated and analyzed in the current study are available from the corresponding author upon reasonable request.

## Abbreviations

ICIs: Immune checkpoint inhibitors
CBDCA: Carboplatin
ABCP: Atezolizumab + carboplatin + paclitaxel + bevacizumab
A + CnP: Atezolizumab + carboplatin + nab-paclitaxel
A + CbP: Atezolizumab + CBDCA + pemetrexed
Nab-PTX: Nab-paclitaxel
ROR: Reporting odds ratio
ADR: Adverse drug reaction
FAERS: FDA Adverse Event Reporting System
CI: Confidence interval
NSCLC: Non-small cell lung cancer
Non-Sq NSCLC: Non-squamous non-small cell lung cancer
PTX: Paclitaxel
PMX: Pemetrexed
OS: Overall survival
PD-1: Programmed cell death protein 1
PD-L1: Programmed death ligand 1
Bev: Bevacizumab
RCT: Randomized controlled trials
FDA: Food and Drug Administration
AKI: Acute kidney injury
DILI: Drug-induced liver injury
PT: Preferred term
