# Supplementary material for: Disproportionality analysis of adverse events in advanced lung cancer treated with atezolizumab plus platinum-based combination chemotherapy
Source: J Pharm Health Care Sci. 2025 Dec 6;12:2. doi: 10.1186/s40780-025-00522-6 (PMC12797384; doi:10.1186/s40780-025-00522-6)
Supplement: Supplementary file 1 — Supplementary Material 1 [file 40780_2025_522_MOESM1_ESM.docx]

Supplementary Table S1.

Regimen-specific reporting odds ratios for hematologic adverse events

| Regimen | Adverse event | Case | ROR | 95% CI |
| --- | --- | --- | --- | --- |
| ABCP | Leukopenia | 12 | 1.99 | 1.13–3.51 |
|  | Neutropenia | 67 | 3.50 | 2.76–4.46 |
|  | Thrombocytopenia | 33 | 2.47 | 1.76–3.48 |
|  | Anaemia | 143 | 6.76 | 5.73–7.98 |
| A+CnP | Leukopenia | 3 | 5.43 | 1.75–16.9 |
|  | Neutropenia | 11 | 6.30 | 3.47–11.4 |
|  | Thrombocytopenia | 5 | 4.09 | 1.70–9.85 |
|  | Anaemia | 11 | 5.64 | 3.11–10.2 |
| A+CbP | Leukopenia | 5 | 8.38 | 3.48–20.2 |
|  | Neutropenia | 21 | 3.62 | 2.36–5.57 |
|  | Thrombocytopenia | 15 | 3.71 | 2.24–6.17 |
|  | Anaemia | 67 | 10.55 | 8.27–13.5 |

Data were extracted from the FAERS database for the period between January 1, 2018, and March 30, 2024. Hematologic adverse events were identified using preferred terms (PTs) from MedDRA version 26.0. The following PTs and corresponding MedDRA codes were used: Leukopenia (PT 10024384), Neutropenia (PT 10029205), Thrombocytopenia (PT 10043554), Anaemia (PT 10002218)

Supplementary Table S2.

Regimen-specific reporting odds ratios for rash and hypersensitivity-related adverse events

| Regimen | Adverse event | Case | ROR | 95% CI |
| --- | --- | --- | --- | --- |
| ABCP | Anaphylactic reaction | 17 | 2.59 | 1.61–4.17 |
|  | Urticaria | 8 | 0.41 | 0.20–0.82 |
|  | Drug hypersensitivity | 3 | 0.10 | 0.03–0.31 |
|  | Erythema multiforme | 37 | 39.61 | 28.62–54.80 |
|  | Stevens–Johnson syndrome | 16 | 8.59 | 5.26–14.04 |
|  | Toxic epidermal necrolysis | 2 | 1.18 | 0.29–4.72 |
|  | Rash | 162 | 2.96 | 2.54–3.46 |
|  | Total | 245 | 2.06 | 1.81–2.34 |
| A+CnP | Anaphylactic reaction | 1 | 1.65 | 0.23–11.76 |
|  | Urticaria | N/A | N/A | N/A |
|  | Drug hypersensitivity | 1 | 0.36 | 0.05–2.59 |
|  | Erythema multiforme | N/A | N/A | N/A |
|  | Stevens–Johnson syndrome | N/A | N/A | N/A |
|  | Toxic epidermal necrolysis | N/A | N/A | N/A |
|  | Rash | 7 | 1.38 | 0.65–2.90 |
|  | Total | 9 | 0.81 | 0.42–1.56 |
| A+CbP | Anaphylactic reaction | 1 | 0.50 | 0.07–3.56 |
|  | Urticaria | N/A | N/A | N/A |
|  | Drug hypersensitivity | 1 | 0.11 | 0.02–0.78 |
|  | Erythema multiforme | N/A | N/A | N/A |
|  | Stevens–Johnson syndrome | 4 | 7.08 | 2.65–18.87 |
|  | Toxic epidermal necrolysis | 1 | 1.94 | 0.27–13.80 |
|  | Rash | 14 | 0.83 | 0.49–1.41 |
|  | Total | 21 | 0.57 | 0.37–0.87 |

Data were obtained from FAERS reports submitted between January 1, 2018, and March 30, 2024. Rash and hypersensitivity-related adverse events were identified using preferred terms (PTs) from MedDRA version 26.0. The following PTs and their MedDRA codes were included: Anaphylactic reaction (PT 10002198), Urticaria (PT 10046735), Drug hypersensitivity (PT 10013700), Erythema multiforme (PT 10015218), Stevens-Johnson syndrome (PT 10042033), Toxic epidermal necrolysis (PT 10044223), Rash (PT 10037844). N/A indicates that no adverse event cases were reported for the specified event-regimen combination.

Supplementary Table S3.

MedDRA preferred terms used to identify renal dysfunction reports

| Preferred term | Adverse event | Case |
| --- | --- | --- |
| 10069339 | Acute kidney injury | 101278 |
| 10002847 | Anuria | 3294 |
| 10059345 | Postrenal failure | 163 |
| 10072370 | Prerenal failure | 771 |
| 10081980 | Subacute kidney injury | 19 |
| 10048302 | Tubulointerstitial nephritis | 11215 |
| 10064848 | Chronic kidney disease | 68314 |
| 10078095 | Chronic kidney disease-mineral and bone disorder | 1032 |
| 10012660 | Diabetic end stage renal disease | 20 |
| 10061835 | Diabetic nephropathy | 1141 |
| 10077512 | End stage renal disease | 16577 |
| 10055171 | Hypertensive nephropathy | 834 |
| 10029159 | Nephrosclerosis | 521 |
| 10062237 | Renal impairment | 41153 |
| 10038428 | Renal disorder | 21295 |
| 10038535 | Renal tubular acidosis | 997 |
| 10046337 | Urate nephropathy | 41 |
| 10051920 | Glomerulonephropathy | 130 |
| 10029164 | Nephrotic syndrome | 2845 |

Data were obtained from FAERS reports submitted between January 1, 2018, and March 30, 2024. Renal dysfunction-related reports were identified using PTs from MedDRA version 26.0. Each PT and its corresponding code used in this analysis is listed in the table along with the number of associated reports. The total number of reports in the analyzed FAERS dataset was 28,136,819. Of these, 271,640 reports (0.97%) were identified as containing at least one of the selected PTs related to renal dysfunction.
